# SAXS Combined with UV-vis Spectroscopy and QELS: Accurate Characterization of Silver Sols Synthesized in Polymer Matrices

**DOI:** 10.1186/s11671-016-1230-2

**Published:** 2016-01-27

**Authors:** Leonid Bulavin, Nataliya Kutsevol, Vasyl Chumachenko, Dmytro Soloviov, Alexander Kuklin, Andrii Marynin

**Affiliations:** Faculty of Chemistry, Taras Shevchenko National University, 60 Volodymyrska str., Kyiv, 0160 Ukraine; Faculty of Physics, Taras Shevchenko National University, 60 Volodymyrska str., Kyiv, 0160 Ukraine; Institute for Safety Problems of Nuclear Power Plants NAS of Ukraine, 12 Lysogirska str., Kyiv, 03680 Ukraine; Joint Institute for Nuclear Research, 6, Joliot-Curie str., Dubna, Moscow region 141980 Russian Federation; Moscow Institute of Physics and Technology, 9 Institutskiy per., Dolgoprudny, Moscow Region 141700 Russian Federation; Problem Research Laboratory, National University of Food Technology, 68, Volodymyrska str., 01601 Kyiv, Ukraine

**Keywords:** SAXS, QELS, UV-vis, Plasmon resonance, Silver nanoparticles, UV-vis spectroscopy

## Abstract

The present work demonstrates a validation of small-angle X-ray scattering (SAXS) combining with ultra violet and visible (UV-vis) spectroscopy and quasi-elastic light scattering (QELS) analysis for characterization of silver sols synthesized in polymer matrices. Polymer matrix internal structure and polymer chemical nature actually controlled the sol size characteristics. It was shown that for precise analysis of nanoparticle size distribution these techniques should be used simultaneously. All applied methods were in good agreement for the characterization of size distribution of small particles (less than 60 nm) in the sols. Some deviations of the theoretical curves from the experimental ones were observed. The most probable cause is that nanoparticles were not entirely spherical in form.

## Background

The size of metal nanoparticles determines optical, catalytic, or biomedical properties of nanosystems and defines limits for applications [1–4]. Despite the increasing interest in the applications of functional nanoparticles, a comprehensive understanding of the formation of nanosystems as well as their precise characterization is still a challenge.

Techniques to detect and characterize nanoparticles fall into two categories: direct, or “real space,” and indirect, or “reciprocal space.” Direct techniques include transmission electron microscopy (TEM), scanning electron microscopy (SEM), and atomic force microscopy (AFM). These techniques can image nanoparticles, directly measure sizes, and infer shape information, but they are limited to studying only a few particles at a time. There are also significant issues surrounding the sample preparation for electron microscopy. In general, however, those techniques can be quite effective for obtaining basic information about a nanoparticle.

Indirect techniques for nanosystem characterization are absorption (ultra violet and visible (UV-vis) spectroscopy) and various scattering methods: quasi-elastic light scattering (QELS), X-rays, or neutron scattering. The techniques that become of greatest relevance to nanoscience are small-angle X-ray scattering (SAXS) and small-angle neutron scattering (SANS) [5, 6]. The advantage of those techniques is that they are able to characterize large numbers of nanoparticles and often do not require any particular sample preparation.

The main aim of the current research was to compare the data obtained by the complex of physical methods for evaluation of various indirect techniques for sol characterization.

## Methods

The silver nanoparticles (AgNPs) were synthesized by reduction of the AgNO_3_ salt using sodium borohydride (NaBH_4_) as reductant. The synthesis of Ag sols was carried out in situ into an aqueous solution of nonionic polymer dextran-graft-polyacrylamide and its anionic derivative [7–9].

The synthesis of AgNPs was performed at the polymer concentration corresponding to dilute polymer solutions.

NaBH_4_ was purchased from “Pharma” (Ukraine).

AgNO_3_ (Sigma Aldrich) was used without additional purification.

### AgNP Synthesis

Reduction of Ag salt was performed at *T* = 60 °C. Molar ratio of AA monomers to Ag^+^ cations was equal to 5. The syntheses were carried out in polymer solutions prepared using deionized water. The pH value of aqueous solutions of nonionic polymer was 5.5 that corresponds to the pH of deionized water. pH value of aqueous solutions of anionic polymers was around 7.33.

Two milliliters of a 0.1 mol L^−1^ AgNO_3_ aqueous solution was added to 5 mL of aqueous polymer solution (*c* = 1.10^−3^ g cm^−3^) and stirred for 20 min. Then, 2 mL of 0.1 mol L^−1^ aqueous solution of NaBH_4_ was added. The final aqueous solution was stirred for 30 min. It turned reddish brown; thus, the formation of AgNPs was indicated.

### Size-Exclusion Chromatography (SEC)

Multidetection size-exclusion chromatography (SEC) analysis of polymers was carried out by using an experimental setup consisting of a LC-10 AD Shimadzu pump (throughput 0.5 mL min^−1^; Nakagyo-ku, Kyoto, Japan), an automatic injector WISP 717+ from Waters (Milford, MA, USA), three coupled 30-cm Shodex OH-Pak columns (803HQ, 804HQ, and 806HQ; Munich, Germany), a multi-angle light scattering detector DAWN F from Wyatt Technology (Dernbach, Germany), and a differential refractometer R410 from Waters. Distilled water containing 0.1 M NaNO3 was used as eluent. Dilute polymer solutions (*c* = 1.10^−3^ g cm^−3^ < c* = 1/[*η*] (Table 1)) were prepared and injected. The intermolecular correlations were then negligible in the analysis of the light scattering measurements. To obtain gyration radii for macromolecules, static light scattering data was analyzed by Zimm method [10].

**Table 1:** Polymer characteristics determined by SEC and potentiometry

Sample	*M*_w_ × 10^−6^, g mol^−1^	*I* = *M*_w_/*M*_n_	*R*_g_, Å	*A*, %
D-g-PAA	1.57	1.81	670	–
D-g-PAA(PE)	1.57	1.81	–	37

### UV-vis Spectroscopy

UV-visible absorption spectra of silver sols were recorded by Varian Cary 50 scan UV-visible spectrophotometer (Palo Alto, CA, USA).

### Quasi-Elastic Light Scattering (QELS)

DLS measurements were carried out using Zetasizer Nano ZS90 (Malvern Instruments Ltd., UK). The apparatus contains a 4-mW He-Ne laser with a wavelength of 632.8 nm, and the scattered light is detected at an angle of 60°.

*SAXS* experiments were carried out on an instrument with a high-intensity microfocus rotating Cu anode X-ray generator in the Laboratory for Advanced Studies of Membrane Proteins (Moscow Institute of Physics and Technology, Dolgoprudniy, Russia), using a standard transmission configuration. An X-ray wavelength of *λ* = 1.54 Å was used, resulting in a momentum transfer *Q* in the range of 0.007–0.2 Å^−1^, where *Q* = (4*π*/*λ*) sin(*θ*/2) and *θ* is the scattering angle. The samples studied were placed in borosilicate capillaries of 1.5 mm diameter and 0.01 mm wall thickness (W. Muller, Berlin, Germany). Water was used as a buffer sample. Center of beam line and conversation channel to value of module *q*-vector was done using silver behenate [11].

## Results and Discussion

The main characteristics of the polymers used as the matrices for in situ AgNP syntheses are drawn in Table 1, where *I* = *M*_w_/*M*_n_, the polydispersity index; *R*_g_, the radius of gyration; and *A*, the chemical charge fraction of polyelectrolytes obtained by alkaline hydrolysis of polyacrylamide.

SEC analysis indicates that polymer samples possess relatively low polydispersity index and display in aqueous solution rather large radii of gyration in agreement with their high average molecular weights. The peculiarities of the molecular structure of the copolymers dextran-graft-polyacrylamide (D-g-PAA) were discussed in [12–14]. These copolymers are star-like polymers, consisting of a compact dextran core and long polyacrylamide arms. As it was previously reported, the branched polymers, due to their more compact internal structure, have higher local concentration of functional groups with respect to their linear analogues [13, 14] that is why they are more efficient matrices for nanosystem fabrication [15].

D-g-PAA copolymer was transformed into polyelectrolyte, referred as D-g-PAA(PE) by alkaline hydrolysis. The process of D-g-PAA hydrolysis was not attended by irrelevant processes (breaking or cross-linking of the macromolecules) [14].

It is evident that saponified polymers contain two types of functional groups: carbamide and carboxylate ones. The pH value of the solutions was equal to 7.33 after the D-g-PAA(PE) sample dissolved in bi-distilled water. Thus, carboxylate groups of polymer were partially hydrolyzed in such conditions. Obviously, the nucleation process occurring just after reductant addition differs for silver ions interacting with carbamide or carboxylate moiety. That could lead to a different size distribution for nanoparticles synthesized in branched nonionic and polyelectrolyte polymer matrices.

In situ syntheses of AgNPs into dilute aqueous solutions of both uncharged (nonionic) and polyelectrolyte branched polymer matrices resulted in rather stable colloids. Our previous attempts to synthesize the stable colloid in anionic linear PAA matrices were not successful; some precipitation has been observed [15].

The sols were studied using indirect technique for nanosystem characterization, namely UV-vis spectroscopy and two scattering methods: QELS and SAXS.

### UV-vis Spectroscopy

**Fig. 1:**
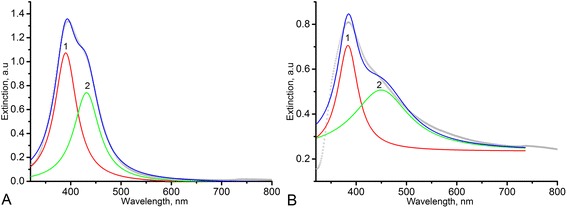
Lorenz multiple peak fit (*blue line*) for Ag NP UV-vis spectra of sol 1 (**a**) and sol 2 (**b**). *Black circle line* experimental spectra, *red line* peak 1, *light green line* peak 2

**Fig. 2:**
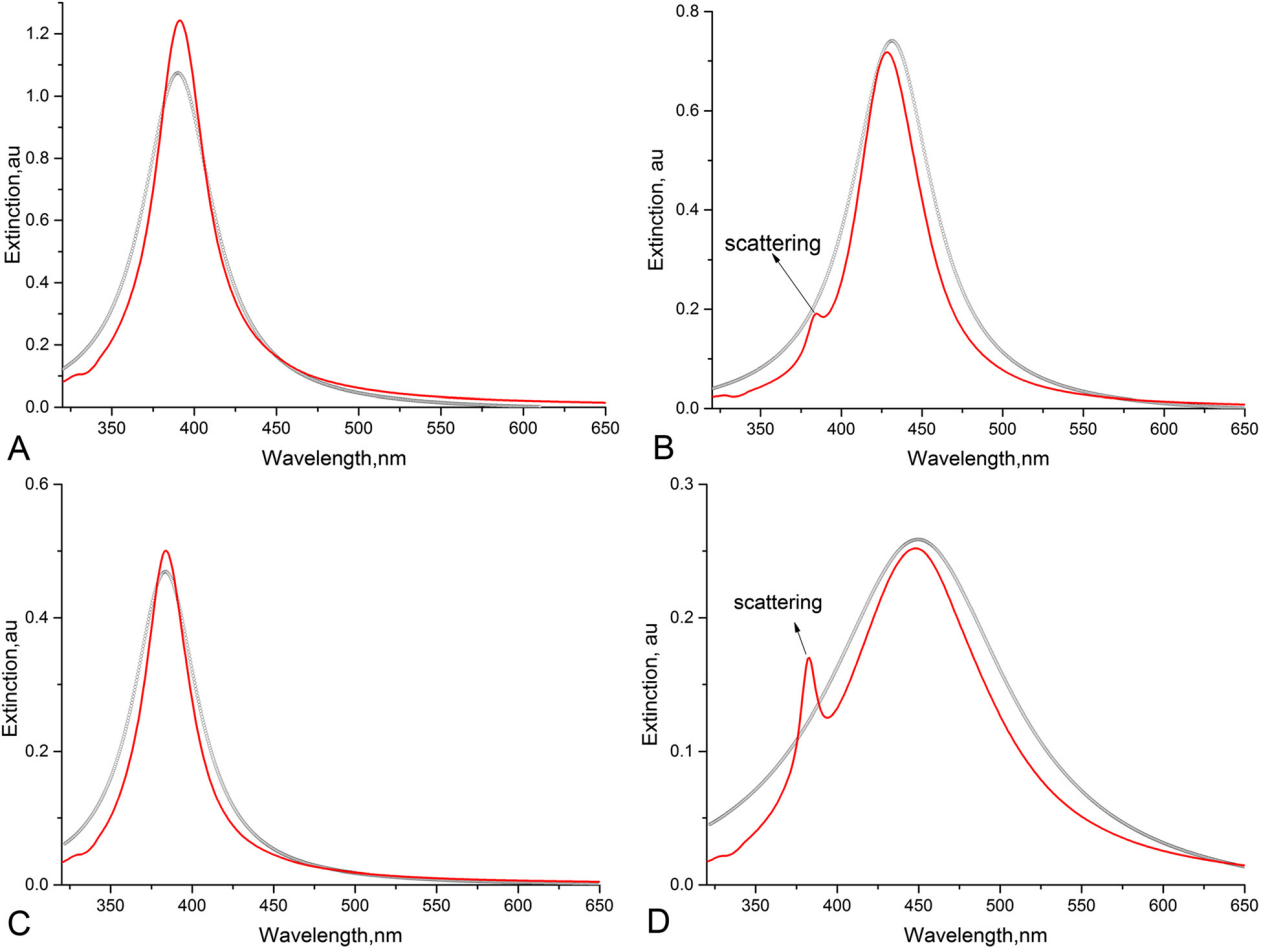
Mie fits of Lorenz curves for sol 1 (**a**—peak 1, **b**—peak 2) and sol 2 (**c**—peak 1, **d**—peak 2). *Black circle lines* Lorenz curves, *red lines* Mie fit

Surface plasmon resonance peak with well-defined shoulders were observed in UV-vis spectra of Ag sols (Fig. 1). For AgNPs (nanosystems-sols) synthesized in D-g-PAA and D-g-PAA(PE), further sol 1 and sol 2, respectively, the extinction maxima were observed at 392 and 386 (Fig. 1a, b, curve 1, peak 1) and shoulders on the plasmon bands at 432 and 451 (Fig. 1a, b, curve 1, peak 2). Such result could be explained by the existence of two size fractions of AgNPs. Experimental extinction curves have been fitted by Lorenz multiple peak fit (OriginLab 9.1) (see Fig. 1a, b; curve 2, 3). In light scattering theory, the most famous theory is likely to be the one published by Gustav Mie in 1908. This theory describes the quasi-elastic interaction between an electromagnetic plane wave and a homogeneous sphere defined by its (arbitrary) diameter and its (arbitrary) complex refractive index. It allows to calculate scattered fields outside the sphere, internal fields, phase relations, and various cross sections. Peaks 1 and peaks 2 have been approximated using MieLab software for spherical homogeneous particles. The algorithm of mathematical analysis and source code are described in detail in [16]. The best fits are represented in Fig. 2. Deviation of theoretical curve takes place for both samples. For both sols, the additional maximum on the theoretical curves (marked as “scattering” on peaks 2) was observed for the second maximum (Fig. 2b–d). These peaks can correspond to scattering contribution to extinction spectra. Absence of this phenomenon on the experimental spectrum for sol 1 and on the Lorenz fit (Fig. 1a) can be explained by overlap of scattering maxima with the peak 1 at the similar wavelength (384 and 386 nm, respectively). A phase retardation of scattering contribution appeared to be more significant for the case of sol 2 (Fig. 2d). It may be related to the presence of the fraction of larger particles in sol 2 in comparison with sol 1. As the result, the shoulder on the experimental spectrum corresponded to scattering could be observed in the range of 380 nm (Fig. 1b). Parameters of Mie approximation, namely sphere size and polydispersity, are represented in Table 2.

**Table 2:** Size characteristics of sols calculated using Mie theory

Sample	Peak 1		Peak 2	
	R, Å	Polydispersity, %	*R*, Å	Polydispersity, %
Sol 1	19	51	*250*	*30*
Sol 2	15	42	*385*	*21*

Some deviations of the theoretical curves from the experimental ones were observed (Fig. 2). The most probable cause is that nanoparticles were not entirely spherical in form, as described in the theoretical model. But the average diameters of AgNPs estimated from the theoretical curves proved to be very close to the ones evaluated from TEM images in our previous work [13].

### QELS Analysis

**Fig. 3:**
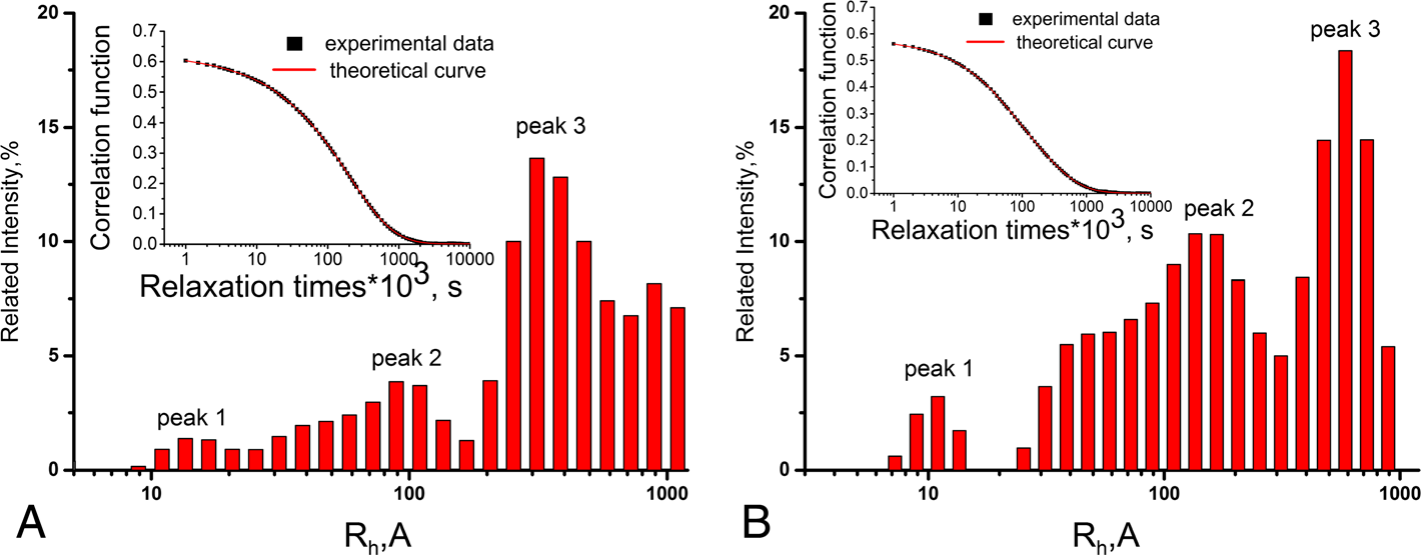
Approximated correlation functions (*inserted graphs*) and intensity-weighted hydrodynamic radii distributions for sol 1 (**a**) and sol 2 (**b**)

Regularized inverse Laplace transform of experimental correlation functions was performed using MathLab code rilt.m (inserted graphs in Fig. 3) [17]. Hydrodynamic radii of particle scatter have been reached from Stokes-Einstein equation:

1 $$ {R}_{\mathrm{h}}=\frac{kT}{6\pi \eta D} $$

where *k*—Boltzmann constant, *T*—absolute temperature, *η*—viscosity, and *D*—diffusion coefficient.

The results of analysis are represented in Fig. 3. Intensity-weighted distributions for both nanosystems have a complicated multimodal shape. The existence of 10–20-Å nanoparticles in sol 1 (peak 1, Fig. 3a) and 7–15 Å in sol 2 (peak 1, Fig. 3b) is evident. The fractions of larger AgNPs of 100–150 Å (peaks 2; Fig. 3a, b) and the aggregates of 200–1000 Å (peaks 3; Fig. 3a, b) are observed in both sols. Statistical analysis of distribution curves is represented in Table 3.

**Table 3:** Statistical analysis of size distribution curves obtained by QELS data analysis

Sample	Peak	*R*_h_, Å (at peak maximum)	St. error, Å
Sol 1	1	16	0.5
	*2*	*95*	*2.5*
	3	310	3
Sol 2	1	11	0.2
	*2*	*153*	*1.2*
	3	603	3.5

The peaks in the range of 200–1000 Å can correspond to the aggregates of AgNPs as well as to the macromolecules of polymer matrices (Table 1). QELS results are in good agreement with the UV-vis results excluding the peak of AgNP aggregates and macromolecules.

### SAXS Data Analysis

**Fig. 4:**
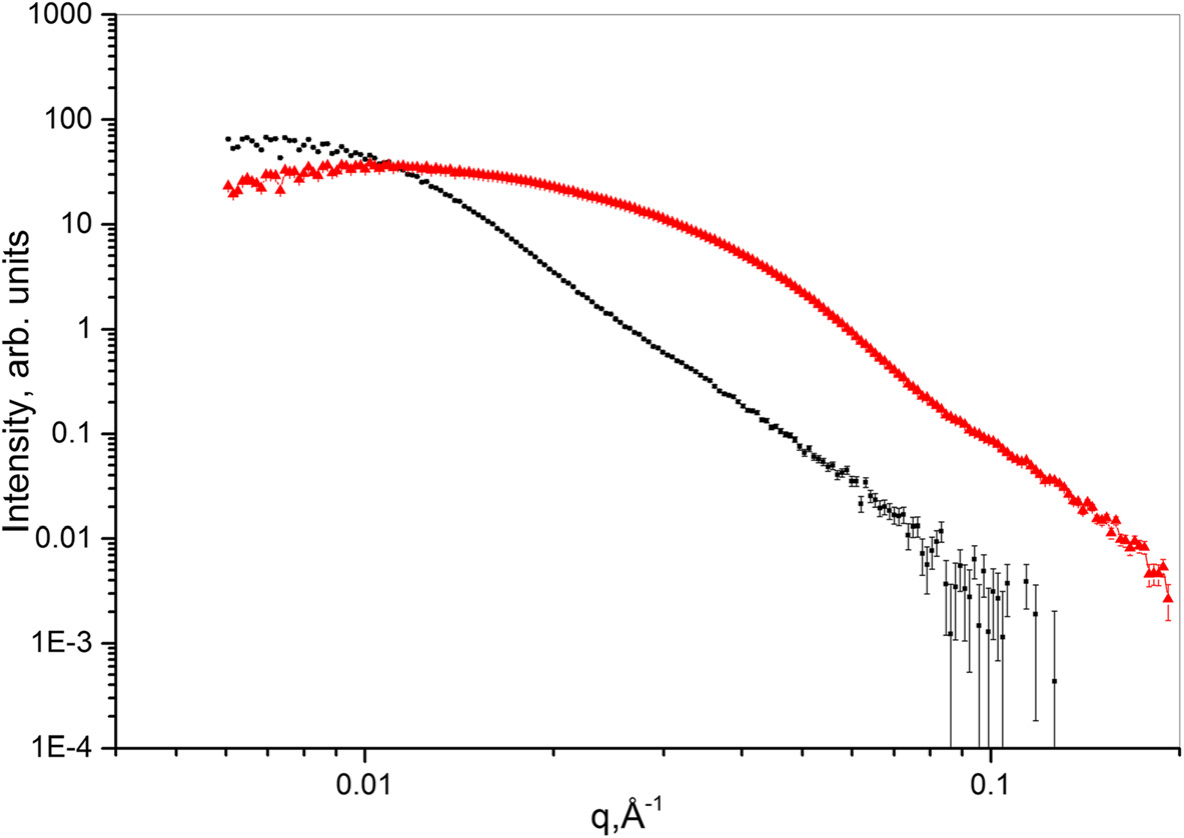
SAXS curves of sol 1 (*black*) and sol 2 (*red*)

The small-angle X-ray scattering curves for sol 1 and sol 2 are represented in Fig. 4. Curves were normalized on sample transmission; scattering from buffer sample (water) was subtracted.

For the analysis of experimental data, the following methods were used: Guinier plot, size distribution function plot, and fitting of the obtained scattering curves. For Guinier plot and for the size distribution function plot, the PRIMUS program from the software package ATSAS was applied [18, 19]. Experimental curve fitting was provided using SASVIEW program [20].

The gyration radii (*R*_g_) of AgNPs in sol 1 and sol 2 were determined using Guinier plot. The influence of structure factor was taken into account. The results of analysis are drawn in Table 4. The third column (Table 4) is shown for evaluation of applicability of Guinier approximation.

**Table 4:** The radii of gyration (*R*_g_) of AgNPs from Guinier plot

Sample	*R*_g_, Å	*qR*_g_ limits
Sol 1	*70 ± 2*	0.79–1.28
Sol 2	*134 ± 33*	0.8–1.3

This function depends both on the particle’s geometry, expressing numerically the set of distances joining the volume elements within a particle, and on a particle’s inner inhomogeneity distribution.

**Fig. 5:**
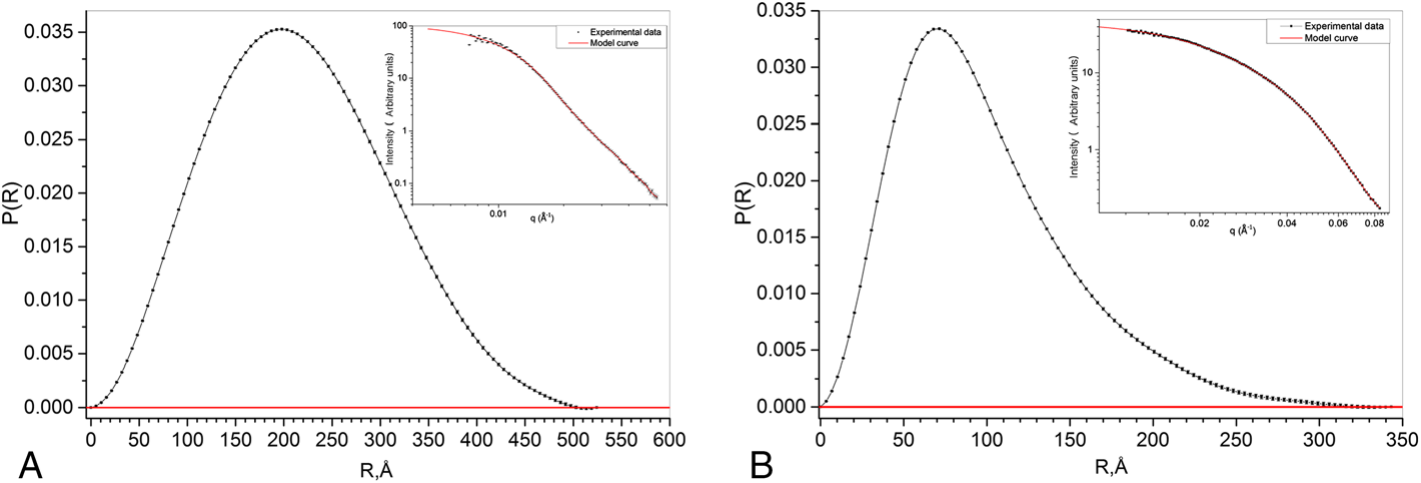
Size distribution function for sol 1 (**a**) and sol 2 (**b**)

For size distribution function analysis, the program PRIMUS of the software package ATSAS was used. The results are represented in Fig. 5a, b for sol 1 and sol 2, respectively. The gyration radius as well as the particle size maximum in both sols are shown in Table 5.

**Table 5:** The gyration radius and maximum particle size in the sols (from size distribution functions)

Samples	*R*_g_, Å	*R*_max_, Å
Sol 1	*78*	342
Sol 2	*166*	524

Here, *R*_max_ is the largest distance between the volume elements within a particle.

**Fig. 6:**
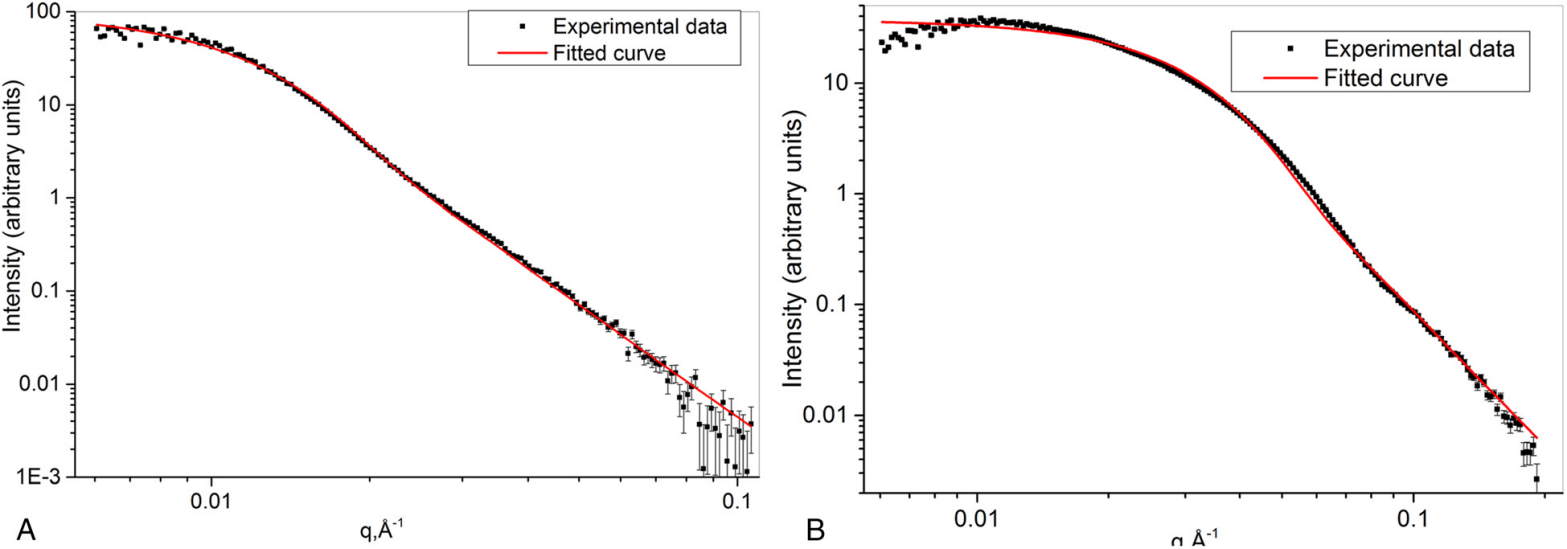
Fitting of the experimental curves, obtained using hard sphere model with polydispersity. sol 1 (**a**), sol 2 (**b**)

Figure 6a, b represents the results of fitting the experimental scattering curves for sol 1 and sol 2, respectively. Sphere model function with polydispersity as a model fitting function was used. Scattering intensity formula for sphere model function is

2 $$ I(q)=\frac{\mathrm{scale}}{V}\cdot {\left(\frac{3V\left(\varDelta \rho \right)\left( \sin (qR)- qRcos(qR)\right)}{(qR)^3}\right)}^2+\mathrm{b}\mathrm{k}\mathrm{g} $$

where scale is a volume fraction, *V* is the volume of the scatterer, *R* is the radius of the sphere, bkg is the background level, and *Δρ* is the contrast [21].

The resulting particle size with a comparison with results obtained by other methods is shown in Table 6.

**Table 6:** Summary results of SAXS analysis

Sample	Guinier plot	Size distribution	Fitting	
	*R*_g_, Å	*R*_g,_ Å	*R*, Å	Polydispersity, %
Sol 1	70 ± 2	78	44	45
Sol 2	134 ± 33	166	118	48

Some fitting inaccuracies occurred on Fig. 6a, b in the range of small *q* values. It can be caused by the interaction between scattering particles in the aggregates. The fit model does not take it into account. However, the dimensions obtained after the fitting of SAXS results are in good agreement with the size characteristics derived by other methods. This fact indicates the correction of the fit model.

Table 6 joins all parameters obtained from SAXS analysis.

SAXS analysis demonstrates monomodal scatterer size distribution in both sols in contrast to QELS and UV-vis spectroscopy, where multimodal particle size distribution is observed. Such contradiction may be caused by the ability of QELS and UV-vis to register large particles or aggregates within the range 300–600 Å. SAXS data analysis is accurate in the limited *q*-range value, 0.02 Å^−1^ < *q* <0.4 Å^−1^. Thus, large particles and aggregates are “invisible” for the *q* values we used.

Size parameters of the nanoparticles estimated correctly by UV-vis, QELS, and SAXS have been marked by italic font within Tables 2, 3, 4, and 5. These values appeared to be close for all techniques. Three different indirect methods also reveal the similar difference in size distribution in nanosystems synthesized in nonionic and anionic branched polymer matrices. The reason for such distinction is the various chemical nature of the polymer template affecting on the nucleation process in the process of nanoparticle formation.

The TEM investigation of silver sols has shown that most AgNPs synthesized in the solution of nonionic branched polymer matrices D-g-PAA had sizes in the range of 8–15 nm. The small number of aggregates was observed too. Silver sols synthesized in branched anionic polymer matrices D-g-PAA(PE) along with NPs have a size of 10–15 nm, i.e., the same as in the sols synthesized in the nonionic polymer matrix. Nanoparticles with a size of 2–5 nm and some large aggregates were observed.

Thus, the present work confirmed the validation of UV-vis spectroscopy and scattering methods for accurate investigation of sols. But UV-vis and SASX are limited for characterization of polydispersed nanosystems and should be used in combination with QELS or TEM.

## Conclusions

The present study proved the efficiency of using branched nonionic and anionic polymers as matrices for the stable silver sols preparation. It was demonstrated that the chemical nature of polymer matrix (uncharged or charged) and the polymer internal structure affect the nanoparticles’ actual control on the sol size characteristics and nanoparticle size distribution in the nanosystems. The analysis of the silver sols was performed using UV-vis spectroscopy, QELS, and SAXS. All methods used were in good agreement for the characterization of size distribution of small particles (less than 60 nm) in the sols. The polydispersity estimated by various methods was comparable. It was shown that for precise analysis of sols synthesized in polymer matrices all these techniques should be used simultaneously. It should be noted that nanoparticle aggregates and macromolecules of the polymer matrix can be characterized only by QELS.
